# Sub-50 nm optical imaging in ambient air with 10× objective lens enabled by hyper-hemi-microsphere

**DOI:** 10.1038/s41377-023-01091-9

**Published:** 2023-02-28

**Authors:** Guangxing Wu, Yan Zhou, Minghui Hong

**Affiliations:** 1 Department of Electrical and Computer Engineering, National University of Singapore, 4 Engineering Drive 3, Singapore, 117576 Singapore; 2 School of Aerospace Engineering, Xiamen University, Xiamen, 361005 China; 3 Present Address: Peng Cheng Laboratory, Shenzhen, 518055 China

**Keywords:** Super-resolution microscopy, Imaging and sensing

## Abstract

Optical microsphere nanoscope has great potential in the inspection of integrated circuit chips for semiconductor industry and morphological characterization in biology due to its superior resolving power and label-free characteristics. However, its resolution in ambient air is restricted by the magnification and numerical aperture (NA) of microsphere. High magnification objective lens is required to be coupled with microsphere for nano-imaging beyond the diffraction limit. To overcome these challenges, in this work, high refractive index hyper-hemi-microspheres with tunable magnification up to 10× are proposed and realized by accurately tailoring their thickness with focused ion beam (FIB) milling. The effective refractive index is put forward to guide the design of hyper-hemi-microspheres. Experiments demonstrate that the imaging resolution and contrast of a hyper-hemi-microsphere with a higher magnification and larger NA excel those of a microsphere in air. Besides, the hyper-hemi-microsphere could resolve ~50 nm feature with higher image fidelity and contrast compared with liquid immersed high refractive index microspheres. With a hyper-hemi-microsphere composed microscale compound lens configuration, sub-50 nm optical imaging in ambient air is realized by only coupling with a 10× objective lens (NA = 0.3), which enhances a conventional microscope imaging power about an order of magnitude.

## Abstract (web summary)

The hyper-hemi-microsphere fabricated by FIB milling can resolve ~50 nm feature by only coupling with a 10× objective lens (NA = 0.3) in virtual imaging mode.

## Graphical abstract

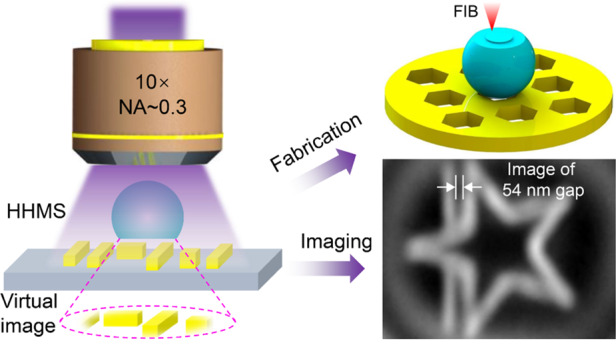

## Introduction

As the semiconductor fabrication techniques rapidly develop, a striking density of nanowires and transistors with sub-100 nm size is integrated on a small chip^1^. In the manufacturing processes of semiconductor chips, the direct inspection of chips is essential for quality control. To discern sub-100 nm features on chips, many tools including scanning electron microscope, atomic force microscope and ptychographic X-ray computed tomography are implemented^2^. However, they have limitations including the requirement of vacuum environment, time-consuming sample preparation and expensive synchrotron radiation source, which is usually not accessible for broad applications. To achieve simple and efficient inspection of chips, optical microscopes working in ambient air are promising alternatives due to its user-friendly and non-destructive characteristics. However, the sub-100 nm features in many key commercial chips, e.g., the central processing unit (CPU) in a computer, have been far beyond the resolution of conventional optical microscopes. Although many super-resolution optical microscopes, such as stimulated emission depletion microscope (STED), stochastical optical reconstruction microscope (STORM), near-field scanning optical microscope and hyperlenses nanoscope^3–7^, have been well developed these years. They still have some restrictions in optical super-resolution imaging, which impede their applications in semiconductor area. For example, the STED and STORM rely on fluorescence labeling. Hyperlenses suffer from severe plasmonic material energy losses and require complicated nanofabrication processes.

Recently, the optical microsphere nanoscope has emerged as a competitive method for the inspection of semiconductor chips due to its nano-imaging ability, label-free and real-time imaging characteristics^8–10^. The nano-imaging mechanism of microsphere has been widely studied^11^. To date, although many advances have been made in optical microsphere nanoscopes^12–14^, the pursuit of higher imaging resolution remains an interesting and challenging subject. The imaging resolution of microsphere nanoscope working in air is associated with two crucial factors, i.e., magnification and numerical aperture (NA) of microsphere^15–17^. If the magnification of the microsphere is insufficient that the enlarged virtual image of an object formed via the microsphere is still beyond the resolution limit of the objective lens used on the nanoscope setup, the object cannot be resolved by the microsphere nanoscope. Recently, a remarkable advance on increasing magnification is realized by using a ball lens with refractive index sufficiently close to 2. Due to the extraordinarily high image magnification, i.e., up to 50×, an ordinary cell phones combined with the ball lens achieves a diffraction-limited resolution ~600 nm^18,19^. Nevertheless, exploring more magnification promotion schemes for imaging resolution beyond diffraction limit is still in demand. The numerical aperture of microsphere restricted by its spherical shape is ≤1 in ambient air. Immersing the microsphere in liquid or solid dielectric surroundings by semi-immersion or full-immersion ways can increase the NA and resolution to certain extents^20–23^. However, this approach causes the contamination of samples and the largest achieved NA is only ~1.65 (ref. ^17^).

**Fig. 1:**
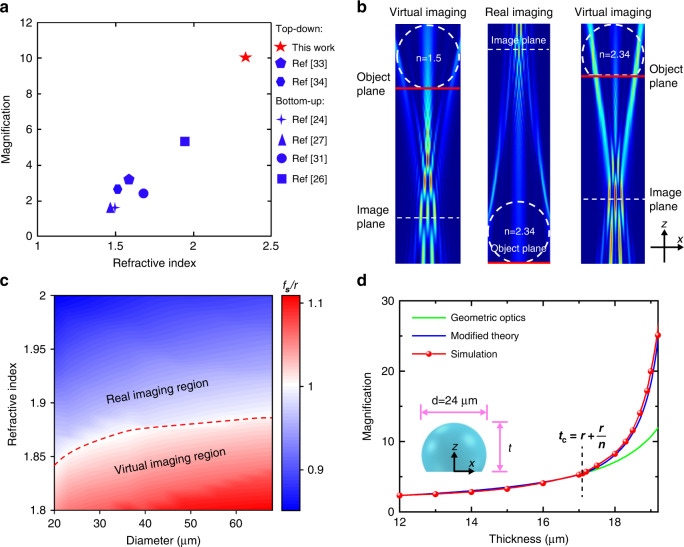
The design and imaging characteristics of HHMSs. **a** Comparisons of magnification and refractive index of PSMLs in literatures and this work. **b** Simulated imaging of *n* = 1.5 microsphere (left, virtual), *n* = 2.34 microsphere (middle, real) and *n* = 2.34 HHMS (right, virtual), respectively. Object planes (red lines), image planes (white dashed lines) and microsphere outlines (white dashed circles) are highlighted in the figure. **c** Relative focal length (*f*_s_/r) of microsphere at different refractive indices and microsphere diameters. **d** Magnification versus HHMS thickness *t*. The green, blue and red plots represent calculation results based on geometric optics theory, modified theory, and simulation results, respectively

In addition to microspheres, plano-spherical microlenses (PSML), including hyper-hemi-microsphere (HHMS) and hypo-hemi-microsphere have also been demonstrated to possess enhanced imaging abilities compared with macroscopic ones^24–26^. HHMS refers to a PSML with shape between a hemi-microsphere and a full microsphere. Unlike microspheres, the geometric shape of PSMLs can be altered to offer different combinations of focal length and object distances, enabling on-demand magnification factors and increased NA. So far, many bottom-up methods have been utilized to fabricate the PSML^27^. In 2009, Lee et al. firstly proposed a chemical method to grow the PSML with calix hydroquinone molecules and demonstrated the nano-imaging ability of PSML. However, the refractive index of calix hydroquinone material is 1.5, which results in a small magnification of the PSML (~1.6×). Later, using the nano-particles self-assembly method to form the HHMS for nano-imaging is widely studied^26,28–31^. Although the refractive indices of nano-particles can be higher than 2, the resulted refractive index of the produced HHMS is below 2 due to inevitable inter-particle spacings^32^. Meanwhile, the assembled microspheres are not robust and easily damaged by an external force or liquid. In terms of top-down methods, the thermal reshaping of colloidal particles and direct laser writing are alternative ways to fabricate the PSML^33–36^. These methods are restricted to some special materials with relatively low melting points, the lens geometry and surface roughness are also not well controlled. None of these reported PSML with visible light transparency possesses an effective refractive index higher than 2. The low refractive index results in a low NA. Moreover, when forming virtual images, the magnification factor of such PSMLs is relatively low. The magnifications and refractive indices of PSML in some reported works are summarized and presented in Fig. 1a. Generally, HHMSs possess higher magnification compared with hypo-hemi-microspheres due to larger thicknesses and thus are preferred.

To overcome these challenges, a top-down method to fabricate the HHMS by subtractive engineering from a high refractive index microsphere is proposed. In this work, the HHMSs made of BTG (*n* ~ 2.34 at 420 nm wavelength) are fabricated and the magnification of a single HHMS as high as 10× is achieved by controlling the height of etching. Experimental results show that the HHMS is able to resolve sub-50 nm features being only coupled with a 10× objective lens due to the increase in the magnification and NA. The imaging contrast of HHMS is also better than that of the microsphere. It is mainly attributed to the flat bottom surface of HHMS and the elimination of Newton’s rings effect. Moreover, a compound micro-lens composed of a HHMS and an adjoining microsphere is utilized to achieve enhanced imaging performance with a larger field-of-view (FOV). The proposed method is suitable for microspheres made of any materials, which promises a high potential to pursue higher NAs for better imaging resolution.

## Results

### Theory

According to the geometric optics theory, the focal point of a microsphere with a refractive index of 2 is at the surface boundary of the microsphere. When a microsphere contacts with sample surface during imaging, if the refractive index of the microsphere is smaller or larger than 2, it works in a virtual or real imaging manner, respectively. Examples of the virtual and real imaging of microspheres are illustrated in Fig. 1b, which are acquired by 2D full-wave simulations. The real imaging is directly simulated using the Lumerical FDTD software, whereas the 2D full-wave virtual imaging simulation requires additional data processing based on the principle of back propagation (see detailed simulation method in Supplementary Materials, Section 1) ^11,37–39^. The sample used for simulation are three 300 nm-spaced electric dipoles oscillating along the *x* direction with respect to the coordinate system shown in Fig. 1b. These dipoles are placed at the bottom boundary of microspheres. The central dipole locates at the central axis of microspheres. The previous researches disclose that the focal length of microsphere is shorter than the geometric optics prediction due to some significant wave optics effects^40,41^. To find the actual critical refractive index of the microsphere such that the focal point just locates at the surface boundary, the focusing light fields of 20–70 μm microspheres are simulated with varying refractive indices by Lumerical FDTD software. The ratios of the focal length *f*_*s*_ to radius *r* of microspheres are presented in Fig. 1c. The red dashed curve indicates the critical refractive index values, at which *f*_s_/(*r* = 1). The critical refractive index value varies with the microsphere diameter and increases from ~1.84 to ~1.89 in the simulated diameter range. In case that a microsphere contacts with the sample surface for imaging, the above critical refractive index can be used to divide the different imaging regimes of the microsphere, i.e., virtual or real imaging, as indicated in Fig. 1c. Specifically, if the microsphere’s refractive index is smaller or larger than the corresponding critical refractive index, it works in a virtual or real imaging regime, respectively. Most previous reported HHMS has a refractive index below the corresponding critical value and works in virtual imaging mode when the HHMS contacts with the sample surface. In such a scenario, the object distance of HHMS is equal to its thickness and decreases with the reduction of thickness *t*, and thus the magnification factor of virtual imaging is impossible to be larger than that of a microsphere without being truncated working in contact mode. The thickness *t* of an HHMS is defined as the distance between the top pole and the flat surface of HHMS, and *d* refers to the diameter of the original microsphere, as shown in the inset of Fig. 1d. To realize a higher magnification, a microsphere with a refractive index larger than the critical value is essentially taken as the primary element to be engineered into HHMS. In this work, commercially available barium titanate glass (BTG) microspheres (*n* ~ 2.34 at 420 nm wavelength) are used to demonstrate the enhanced nano-imaging capacity of HHMS.

To design the HHMS, the most important issue is to determine the thickness of HHMS, which affects both magnification and NA. According to the geometric optics theory, the relationship between magnification *β* and thickness *t* for a HHMS can be calculated as (see Section 4.4 of ref. ^42^).

1 $$\beta = \frac{n}{{\left( {n_0 - n} \right)\frac{t}{r} + n}}$$

where *r* is the radius, *n* and *n*_0_ the refractive indices of HHMS and environment, respectively. This formula describes the case when the gap between HHMS and sample surface is zero. The magnification of a HHMS (*d* = 24 µm, *n* = 2.34) versus the thickness is calculated with Eq. (1) and shown in Fig. 1d with a green curve. Generally speaking, the parameters of micro/nano-scaled lens calculated by geometric optics theory is not accurate. Recently reported work based on rigorous solution of the Maxwell equations has also confirmed the deviation^18^. It is because the geometric optics theory does not consider the size related wave optics effects, such as diffraction and scattering. As a comparison, the 2D full-wave virtual imaging simulations based on the principle of back propagation are conducted for HHMSs with varied thicknesses (See simulation method in Supplementary Materials, Section 1)^11,37–39^. The sample used for simulation are three 300 nm-spaced electric dipoles oscillating along the *x* direction with respect to the coordinate system shown in Fig. 1d. These dipoles are placed at the bottom boundary of HHMSs with the central dipole positioned at the central axis of HHMSs. In this simple case, the coherence of electric dipoles used in the simulation has limited influence on magnification evaluation (see more details in Supplementary Materials, Section 1). A typical virtual imaging result of a HHMS is illustrated in Fig. 1b (see more simulated imaging results in Fig. S3 of Supplementary Materials). The magnifications extracted from simulated imaging results are plotted in Fig. 1d with a red curve. It can be found that the magnification calculated by geometric optics is well consistent with the simulated magnification when the thickness of HHMS is smaller than a critical value *t*_c_. However, as the thickness of HHMS exceeds the critical value *t*_c_, the deviation between the two magnifications significantly rises with the increase in thickness. Intriguingly, the value of the critical thickness *t*_c_ is close to *r* + *r*/*n*, which is just the critical thickness for total internal reflection of light emitting from the bottom surface of HHMS with 90° propagation angle (see detailed analysis in Supplementary Materials, Section 3). The fantastic coincidence may imply some size dependent nano-imaging mechanism and require further theoretical research, which is not the major focus of this work.

To provide more accurate predictions on magnification without case-by-case simulation, the effective refractive index *n*_eff_ of HHMS is proposed to modify the geometric optics formula. The *n*_eff_ of HHMS is defined in Eqs. (2) and (3):

2 $$n_{{{{\mathrm{eff}}}}} \,= \,\left\{ \begin{array}{ll}n,\quad t \le t_{{{\mathrm{c}}}}{{{\mathrm{,}}}}\,t_{{{\mathrm{c}}}} = r + r/n\\ n + \left( {n_{{{{\mathrm{seff}}}}} - n} \right)\left( {\frac{{t - t_{{{\mathrm{c}}}}}}{{f_{{{\mathrm{s}}}}{{{\mathrm{ + }}}}r - t_{{{\mathrm{c}}}}}}} \right),\,f_{{{\mathrm{s}}}} \ge t \,> \,t_{{{\mathrm{c}}}}\end{array} \right.$$

3 $$n_{{{{\mathrm{seff}}}}} = n_0\left( {f_{{{\mathrm{s}}}} + r} \right)/f_s$$

where *n*_seff_ and *f*_*s*_ are the effective refractive index and simulated focal length of the microsphere, which has the same diameter and refractive index with HHMS (see more details about *n*_seff_ and *f*_s_ in Supplementary Materials, Section 2). *f*_s_ measures the distance between the center of microsphere and the focal point, which is the point with the maximum intensity in the nanojet. The effective refractive index *n*_eff_ of HHMS is formulated based on a fact that a thin HHMS well follows the geometric optics norms while it gradually behaves more like a full microsphere with the increase in thickness. Replacing the nominal refractive index *n* of HHMS with its effective refractive index *n*_eff_, Eq. (1) can calculate the magnification of HHMS more accurately. In the design of HHMS composed compound lens (HHMS-CL), the effective refractive index *n*_eff_ also promotes the calculation accuracy for parameters (see more details in Section 6 of the Supplementary Materials). As shown in the blue curve in Fig. 1d, the magnification calculated with the effective refractive index modified theory almost coincides with the simulation results, which demonstrates the effectiveness of the modified theory and can be applied for the design of HHMSs and HHMS-CLs.

Given the fixed number of pixels in an CCD, a higher magnification is preferred in optical nano-imaging to reveal more details of samples. However, the thickness also affects the NA and thus the resolution of HHMS. For HHMSs with different refractive indices, the NA of HHMS maintains the maximum value which is equal to refractive index *n* in the thickness range from *r* to *r* + *n/r*. When the thickness is larger than *r* + *n/r*, NA decreases with thickness due to the total internal reflection (see detailed analysis in Supplementary Materials, Section 3). Therefore, the pursuit of even higher magnification is at the expense of sacrificed imaging resolution. Since both the magnification and NA of HHMS affect the resolving power of the optical HHMS nanoscope, it should be well balanced between these two factors by selecting the proper thickness. In this work, HHMSs with magnifications between 5 and 10× are designed and applied for experiments.

### Fabrication and characterization

**Fig. 2:**
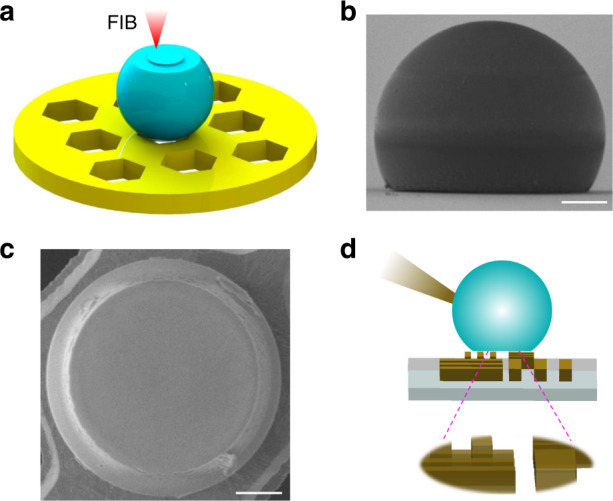
The fabrication and characterization of HHMSs. **a** Schematic of fabricating HHMS by FIB milling. Side view (**b**) and bottom view (**c**) of an HHMS fabricated by FIB. Scale bars: 5 μm. **d** Schematic of HHMS nano-imaging in virtual imaging mode

A schematic of using the FIB milling to fabricate the HHMS is displayed in Fig. 2a. Microspheres are randomly dispersed onto a copper TEM grid, which possesses good electrical conductivity and thus alleviates charge accumulation on the HHMS. Microspheres stuck in the holes of the TEM grid are used. The focused ion beam is applied to remove the material of microsphere layer-by-layer. The thickness of HHMS can be monitored by viewing the milled surface with measurements, whose diameter is used to calculate the thickness of HHMS based on geometric relationships. In principle, the HHMS can be designed to achieve different magnifications by controlling its thickness as analyzed in Fig. 1d. The FIB setup with nanoscale accuracy allows for precise targeted thickness. To achieve high fabrication accuracy, measures to reduce charge accumulation on HHMS are necessary, such as using single scanning mode of FIB and SEM during the fabrication. A typical sample fabricated by this method is shown in Fig. 2b, c. The SEM images prove that the HHMS is fabricated successfully with a smooth surface, which is important for optical imaging. Certainly, if microspheres made of piezoelectric materials or materials with tunable refractive index are used to fabricate the HHMS, it could greatly reduce the accuracy requirement of the fabrication process. In such cases, tuning the refractive index or geometric shape of HHMS can compensate for the effect of thickness deviation to achieve optimized imaging status. After the HHMS is made, a needle tip is used to transfer the HHMS from the TEM grid to sample surfaces for imaging. Schematic of using the fabricated HHMS for nano-imaging in virtual imaging mode is shown in Fig. 2d. The HHMS is rotated such that the flat surface is oriented toward the sample. The HHMS is then fixed on a needle tip with glue. The needle tip is mounted on a high-precision translation stage, with which the HHMS is easily relocated to any area of interest for subsequent optical imaging. In the experiment, it is preferred to maintain a distance between HHMS and sample surface to avoid damage or contamination to the sample surface when the HHMS is moved. Certainly, for a sample with smooth surfaces, such as the semiconductor chips, this system is capable to image a large area by scanning.

### Imaging resolution and magnification of HHMS

**Fig. 3:**
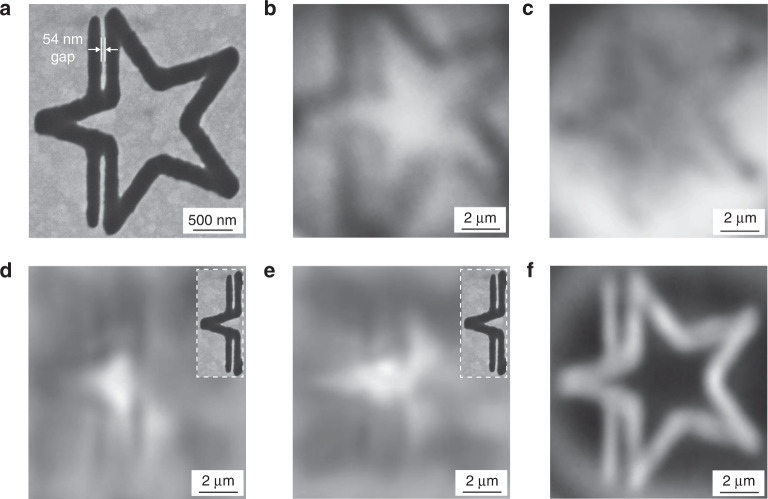
Imaging comparisons among microspheres and HHMS. Star pattern with a 54 nm gap imaged by **a** SEM, **b** a 23 μm borosilicate glass microsphere (*n* ~ 1.52) in air, **c** a 23 μm BTG microsphere (*n* ~ 2.34) in air, **d** an 8 μm BTG microsphere (*n* ~ 1.9) immersed in oil (*n* ~ 1.34), **e** an 8 μm BTG microsphere (*n* ~ 2.2) immersed in oil (*n* ~ 1.5) and **f** a BTG HHMS (*n*~2.34) in air. Insets in (**d**) and (**e**): the corresponding SEM images of the region imaged by microspheres

The resolution of microsphere over macroscopic plano-spherical lenses has been proved in the previous work ^21^. However, the resolution comparison between HHMS and microsphere is still not widely known. In experiments, an HHMS fabricated from a 23.2 μm BTG microsphere (*n* ~ 2.34) working in the air is compared with microspheres working in both air and liquid immersion environments. The thickness of the HHMS is selected to achieve the largest magnification within the maximum NA range. According to theoretical analyses, the thickness of the microsphere should be within ((1 + 1/*n*)*r*, i.e., 16.6 μm. The measured thickness of the fabricated HHMS is ~16.5 μm, which meets the above requirement. The imaging sample for testing is a star pattern with a 54 nm gap on two of its edges. It is fabricated on a silicon substrate with Au coating by FIB, as illustrated in Fig. 3a.

Firstly, a 23 μm borosilicate glass microsphere (*n* ~ 1.52), a 23 μm BTG microsphere (*n* ~ 2.34) and the HHMS are employed to image the star pattern in air environment coupled with a 20× objective lens (NA~0.45). The imaging results are displayed in Fig. 3b, c, and f. The outline of the star pattern can be observed in the virtual image acquired by the borosilicate glass microsphere as shown in Fig. 3b. However, the nano-gap between two separated lines in Fig. 3b is less pronounced compared with that shown in Fig. 3f, which is captured by the HHMS. In Fig. 3c, the image obtained via the BTG microsphere is a real image taken from a plane above the microsphere. It is also hard to discern the real structure in the two edges due to the poor imaging contrast in the real imaging mode. The refractive index of the BTG microsphere and the HHMS is the same, however, the two separated lines can be clearly resolved by the HHMS, which demonstrates better resolution of the HHMS compared with microspheres in air environment. It mainly attributes to the larger NA of HHMS. The NA of the HHMS is ~2.34, which is larger than those (NA~1) of the two microspheres in ambient air and thereby can collect more information on fine structures. Magnifications of the borosilicate glass microsphere, BTG microsphere and HHMS is ~4.8×, ~4.3× and ~4.8× estimated from Fig. 3b, c, and f. Due to larger magnifications of borosilicate glass microsphere and the HHMS, the image quality of Fig. 3b, f is better than Fig. 3c. Higher magnification benefits the image quality by utilizing more pixels on the CCD camera sensor to display the image. Besides, due to the flat surface nature of the HHMS, Newton’s rings do not exist in the virtual image^43^, and thus the imaging contrast of Fig. 3f is much better.

To date, the optimal resolution of microsphere nano-imaging is obtained by 4–10 μm liquid immersed microspheres^11,16^. A direct comparison between the optimal imaging case of microspheres and the HHMS would be useful to benchmark the imaging performance. Here, virtual images of the star pattern acquired by an 8 μm *n* ~ 1.9 BTG microsphere immersed in *n* ~ 1.34 oil and an 8 μm *n* ~ 2.2 BTG microsphere immersed in *n*~1.5 oil are presented in Fig. 3d, e, respectively (see more imaging results in Section 4 of the Supplementary Materials). Refractive indices of immersion medium are selected to achieve optimal refractive index ratio of the microsphere’s refractive index relative to the immersion medium, i.e., around 1.4–1.75 (ref. ^21^). As the FOV of an 8 μm BTG microsphere cannot cover the whole star pattern due to its small size, only a small region of the star pattern is visible for imaging under the microsphere. As such, the smallest feature of 54 nm gap is chosen as the region for immersed BTG microsphere imaging to compare their resolving power with HHMS. The corresponding SEM images of this imaging region are displayed in the insets.

As shown in Fig. 3d, e, the 54 nm gap between two lines in the star pattern is discerned in both virtual images, which indicates that the two immersed microspheres can resolve this feature owing to reduced size and increased NA in the liquid immersion environment. However, compared with the SEM image of the star pattern in this region, significant distortion is observed in the two virtual images. There is an abnormal magnification of the gap. The feature size ratio between line width and gap width in the two virtual images is obviously different from that shown in the SEM image. On the contrary, such feature sizes ratio in the virtual image produced by HHMS is similar to that in the SEM image as shown in Fig. 3f. HHMS can resolve the pattern with significantly higher fidelity, which is very important in practical applications. Besides, HHMS can maintain high resolution in a bigger FOV than liquid immersed 4–10 μm BTG microspheres. The imaging contrast of HHMS is also better. Since the HHMS realizes high NA and resolution in air environment, it can avoid contamination on sample induced by immersion liquid. Therefore, although the resolving power of liquid immersed 4–10 μm BTG microspheres is comparable with HHMS in this case, the HHMS still shows several advantages. Certainly, the current HHMS technology also has its own disadvantages. The proposed nanoscale fabrication method for the HHMSs is expensive and yet impractical for massive production, which may impede its extensive applications. As a comparison, the liquid immersed BTG microspheres are advantageous in this aspect. BTG microspheres are commercially available at affordable prices with mature mass production methods, as such, they can be used in large quantities for nano-imaging applications. Therefore, to further promote the application competence of the HHMS technology, it is necessary to advance the fabrication approach towards being more efficient and low-cost.

**Fig. 4:**
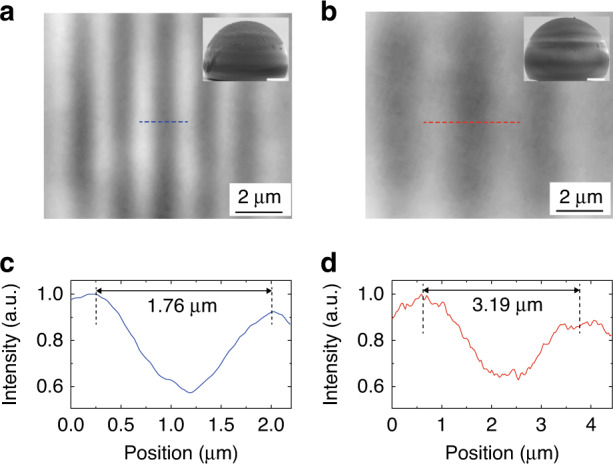
Customizable magnification of HHMSs. Images of a blu-ray disc captured by *n* ~ 2.34 HHMSs with (**a**) *d* = 23.2 μm, *t* = 16.5 μm and (**b**) *d* = 23.7 μm, *t* = 17.9 μm. The insets display the side-view SEM images of the two HHMSs. Scale bar in insets: 5 μm. **c**, **d** Intensity profiles along blue and red dashed lines in (**a**) and (**b**), respectively

Magnification is critical in optical imaging. The typical magnification of a single microsphere is below 5× and hard to be tuned. While the magnification of HHMS with a refractive index higher than the critical value can be easily changed in a wide range by controlling the ratio of thickness to diameter. To demonstrate the tunable magnification of HHMS, two HHMSs with different ratios of thickness to diameter are fabricated and presented in the insets of Fig. 4a, b. The thicknesses and diameters of the two HHMSs are *t* = 16.5 μm, *d* = 23.2 μm and *t* = 17.9 μm, *d* = 23.7 μm, respectively. The corresponding ratios of thickness to diameter are 0.71 and 0.76, respectively. According to Eq. (1), larger ratios of thickness to diameter can realize higher magnification. Their imaging performances are checked with the same Blu-ray disc, and imaging results are shown in Fig. 4a, b, respectively. The period of the stripe in the Blu-ray disc is 320 nm. After being magnified by the two HHMSs, the period becomes 1.76 and 3.19 μm as shown in Fig. 4c, d, respectively. Therefore, the magnifications of these two HHMSs are ~5.5× and ~10×, respectively, which are consistent with the theoretical calculations. As such, the magnification of HHMS can be tailored flexibly. The available large magnification may also benefit the resolution of HHMS assisted nanoscope. If the magnification of the HHMS is insufficient that the size of enlarged virtual image produced by the HHMS is beyond the optical diffraction limit, the objective lens of HHMS assisted nanoscope is unable to resolve the virtual image. The full potential of imaging resolution of HHMSs can be completely exhibited with high magnifications whereas microspheres may be restricted by their insufficient magnifications. (see more analyses in Section 4 of the Supplementary Materials).

There are other methods that can increase the magnification, such as using microspheres compound lens or ball lens with refractive index near the critical value (see detailed comparison between them and HHMS in the Supplementary Material, Section 5)^18,19,44^. However, microspheres compound lens can increase magnification whereas its super-resolution capability is the same as the bottom microsphere. 50× magnification of sphere with refractive index near the critical value has been demonstrated recently but it is not easily tuned at will. The HHMS could be a competitive alternative to solve these issues. The contrast is slightly lower when the HHMS is designed for higher magnification, as illustrated in the intensity profiles of Fig. 4c, d. It is because the increase of magnification sacrifices the NA, which decreases from 2.34 to 1.94 as shown in Fig. S5b. Nevertheless, the NA of HHMS is still larger than microspheres in air and most oil immersion environments.

### Increasing the field-of-view with HHMS-CLs

**Fig. 5:**
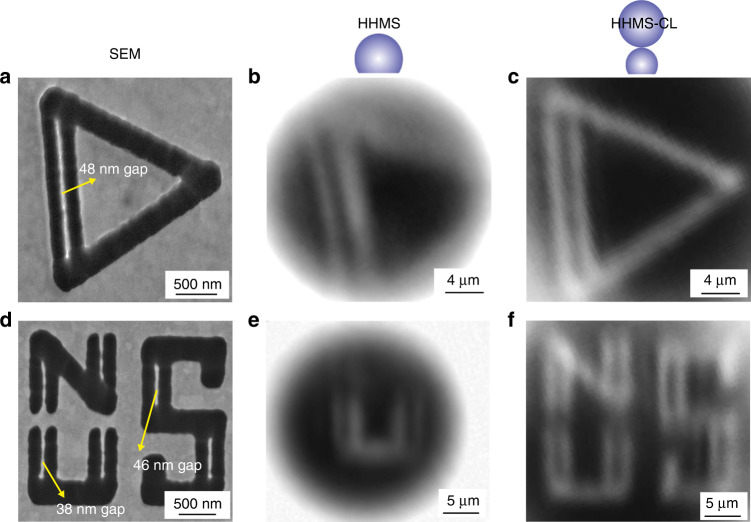
Imaging comparison between the HHMS and HHMS-CL. Triangular pattern and “NUS” letters imaged by (**a**) and (**d**) SEM, **b** and **e** single HHMS being coupled with a 10× objective lens, **c** and **f** HHMS-CL being coupled with 10× objective lens. The minimum feature sizes in the two patterns are 48 and 38 nm, respectively

Typically, a HHMS with a higher magnification has a smaller FOV. As shown in Fig. 5b, a triangle pattern on a silicon substrate with Au coating is imaged by an HHMS, whose magnification is ~10×. The SEM image illustrates that the triangle pattern occupies a ~2 μm × 2 μm area as displayed in Fig. 5a. Only a part of the pattern can be observed in Fig. 5b by single HHMS being coupled with a 10× objective lens (NA ~ 0.3). It is because the high magnification of the HHMS results in a big emission angle of light exiting from the spherical surface of the HHMS. As the emission angle is larger than the collection angle of the objective lens above the HHMS, not all light from the pattern through the HHMS can be collected by the objective lens. Thus, only the central part of the magnified virtual image is captured at the camera plane. To increase FOV and magnification, the microsphere compound lens configuration was previously reported^44,45^. The HHMS is also compatible with the compound lens configuration and may benefit from it.

To demonstrate this, the above HHMS (*d* = 23.7 μm, *t* = 17.9 μm, *n* ~ 2.34) is used to construct the compound lens together with a ~95 μm borosilicate glass microsphere (*n* ~ 1.52). There is no special constraint on the material of the top microsphere as long as the microsphere enables the HHMS-CL to work in virtual-real imaging mode^44^. The diameter of the borosilicate glass microsphere is designed to maintain the 10× magnification but increase the FOV. Details about designing the HHMS-CL are presented in Supplementary Materials, Section. 6. The image of the triangle pattern captured by the HHMS-CL coupled with a 10× objective lens is presented in Fig. 5c. The whole pattern of 2 μm × 2 μm is then completely displayed in one image. The increase in FOV is further verified using a square-shaped “NUS” letters sample. Images captured by SEM, single HHMS and the HHMS-CL are presented in Fig. 5d–f. It can be found that the FOV of the HHMS-CL is around three times larger than that of a single HHMS. No significant change in the resolution of the compound lens is observed. Sub-50 nm features in the two patterns are all discerned by both the single HHMS and the HHMS-CL coupled with 10× objective lens. Therefore, a well-designed compound lens can maintain the magnification and resolution of HHMS, and at the same time improves its FOV. This is a unique benefit of the HHMS-CLs in optical nano-imaging for sub-50 nm features.

### Benchmarking imaging performance

**Fig. 6:**
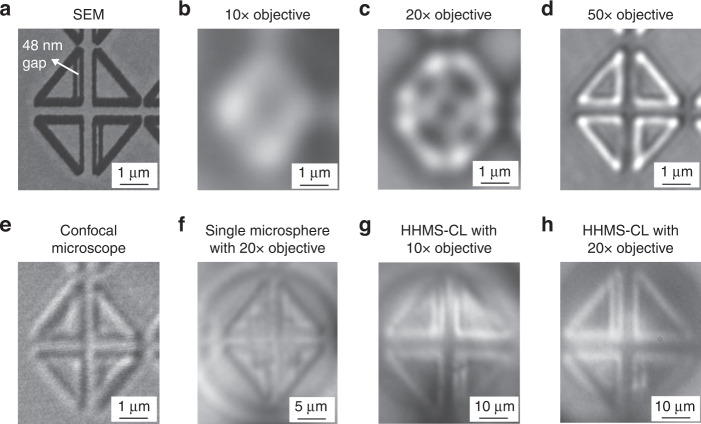
Imaging comparisons among different optical microscopy techniques. A rhombus-shaped pattern with ~48 nm gap is imaged by (**a**) SEM, **b**–**d** conventional optical microscope with 10×, 20× and 50× objective lenses, **e** confocal microscope, **f** single borosilicate glass microsphere nanoscope, **g**, **h** HHMS-CL coupled with 10× and 20× objective lenses, respectively

To benchmark the hyper-hemi-microsphere nano-imaging with the conventional optical microscope, confocal microscope, and single microsphere nano-imaging, a rhombus-shaped pattern with a ~48 nm gap as shown in Fig. 6a is selected as the sample. When the magnification power of objective lenses in a conventional optical microscope increase, more details can be resolved. However, even if a 50× objective lens with 0.8 NA is employed, the sub-50 nm gap structure as displayed in Fig. 6d is not discerned. The confocal microscope with a 100× objective lens (NA ~ 0.9) and a borosilicate glass microsphere nanoscope being coupled with a 20× objective lens (NA ~ 0.45) are also unable to resolve the sub-50 nm gap inside the pattern, as displayed in Fig. 6e, f. The sub-50 nm gap can be resolved by the HHMS-CL being coupled with only a 10× objective lens (NA ~ 0.3), as shown in Fig. 6g, which is attributed to the large magnifying power of the HHMS-CL. Certainly, if a 20× objective lens works with the HHMS-CL, the image quality can be further improved as presented in Fig. 6h. These results prove that the HHMS-CL with a higher magnification and NA exhibit superior resolving power to the conventional optical imaging techniques. Optical microscope using an objective lens with a low magnification power and small NA can be significantly upgraded for sub-50 nm imaging in air, which provides a great promise for extensive applications.

### Semiconductor applications

**Fig. 7:**
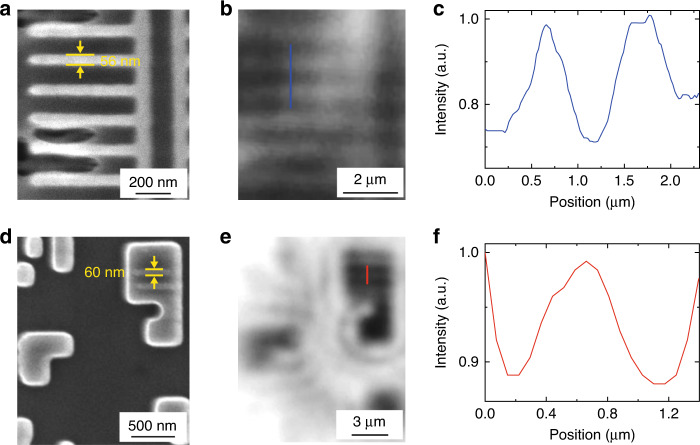
Semiconductor applications of the HHMS. Metal wires and functional devices in a commercial CPU chip are imaged by (**a**) and (**d**) SEM, and (**b**) and (**e**) HHMS. **c**, **f** Intensity profiles along the blue and red lines in (**b**) and (**e**)

To demonstrate the practical applications of our developed HHMS optical nano-imaging techniques, metal wires and functional devices in a commercial CPU chip are used as samples for testing. The smallest width of metal wires in the chip is around 50–60 nm as shown in the SEM image of Fig. 7a. The image of these metal wires captured by a HHMS being coupled with 20× objective lens is presented in Fig. 7b. Metal wires and gaps between them are well resolved as displayed in Fig. 7c. Functional devices on the bottom layer of the chip can also be observed as shown in Fig. 7d, e. Specifically, features with 60 nm linewidth on the “knife-shaped” device are discerned as shown in the intensity profiles displayed in Fig. 7f. These imaging results for the CPU chip demonstrate the potentials of the proposed technique for semiconductor industrial applications.

## Discussion

In summary, a versatile top-down method is proposed to fabricate the HHMSs by subtractively engineering a microsphere with the FIB milling. This method is suitable for microspheres made of any materials, which is superior to other fabrication methods. High refractive index HHMSs (*n* ~ 2.34) are fabricated and experimentally demonstrated with better imaging resolution than a single microsphere in air. The imaging fidelity and contrast of HHMS are better than those of liquid immersed high refractive index microspheres when they resolve ~50 nm feature. HHMSs with tunable magnifications from 5× to 10× are enabled by the FIB precision fabrication. Benefiting from the high magnification and high NA, sub-50 nm features can be discerned by a hyper-hemi-microsphere only coupled with a 10× objective lens in ambient air, which promote the imaging power of a conventional microscope with the same objective lens about an order of magnitude. Furthermore, the HHMS-CL is demonstrated to effectively increases the FOV of a single HHMS without affecting the resolution. High refractive index HHMS and compound lens provide possibilities for easy, fast, and low-cost inspection in semiconductor chip manufacturing and other fields.

## Materials and methods

### Materials

Microspheres used in this works include 20–27 μm BTG microspheres (BTGMS-ND2.2–4.35 20–27 μm -5g), borosilicate glass microspheres (S-BSGMS-2.2 87–95 μm - 1 g), 5–22 μm *n* ~ 1.9 BTG (BTGMS-4.15 5–22 μm - 0.1 g) microspheres and 5–22 μm *n* ~ 2.2 BTG microspheres (BTGMS-ND2.2–4.35 5–22 μm - 0.1 g) are bought from Cospheric company. The 23 μm borosilicate glass microspheres (Catalog Number: 9020) are bought from Thermo Fisher Scientific. The immersion oils are bought from Cargille Pte Ltd (Refractive index liquids Series: AAA, *n*_D_ = 1.3400 ± 0.0002 and Series: A, *n*_D_ = 1.5000 ± 0.0002). 50 nm Au thin film is deposited on silicon wafer to fabricate imaging samples, including star pattern, triangular pattern, “NUS” letters and rhombus-shaped pattern. The deposition is completed by E-beam evaporator (E-Beam Evaporator-AJA UHV). The commercially available CPU chip used in this work is Intel Xeon E5540 processor manufactured with 45 nm technology. The metal wires and devices in the CPU chip are prepared by chemical etching for imaging.

### Experimental setup

The FIB-SEM machine used in this work is produced by TESCAN company. The FIB Resolution is <2.5 nm at 30 kV. An upright optical microscope working in reflected mode is employed for imaging characterization. The illumination light source is a LED, whose central wavelength is ~420 nm measured by a spectrometer (Ocean FX VIS-NIR Spectrometer). Objective lenses used in the optical microscope include “Nikon TU Plan Fluor 10×/0.30 WD 15”, “Nikon TU Plan Fluor 20×/0.45 WD 4.5” and “Nikon TU Plan Fluor 50×/0.8”. The product model of the commercial confocal microscope used for imaging performance comparison is “Nikon ECLIPSE Ni”, which works with a 0.9 NA objective lens (Nikon TU Plan Apo EPI 100×/0.9).

### Numerical simulations

Focusing fields of microspheres are simulated by Lumerical FDTD software with 2D models. The virtual imaging simulation is conducted with Lumerical FDTD software and MATLAB software.

## Supplementary information

Supplementary information

## Data Availability

The data that support the findings of this study are available from the corresponding author upon reasonable request.
