# Supplementary material for: Sub-50 nm optical imaging in ambient air with 10× objective lens enabled by hyper-hemi-microsphere
Source: Light Sci Appl. 2023 Feb 28;12:49. doi: 10.1038/s41377-023-01091-9 (PMC9974943; doi:10.1038/s41377-023-01091-9)
Supplement: Supplementary file 1 — Supplementary information [file 41377_2023_1091_MOESM1_ESM.docx]

**Supplementary Information for**

**“Sub-50 nm Optical Imaging in Ambient Air with 10× Objective Lens Enabled by Hyper-hemi-microsphere”**

Guangxing Wu ^1^, Yan Zhou ^1,†^ and Minghui Hong ^1,2*^

1. Department of Electrical and Computer Engineering, National University of Singapore, 4 Engineering Drive 3, Singapore 117576, Singapore

2. School of Aerospace Engineering, Xiamen University, Xiamen 361005, China

^†^The current address of Yan Zhou is Peng Cheng Laboratory, Shenzhen 518055, China

^*^Contact details of corresponding author Email: elehmh@nus.edu.sg

Phone: (+65) 6516 1636

The official email addresses of Guangxing Wu: e0444191@u.nus.edu

The official email addresses of Yan Zhou: zhouy05@pcl.ac.cn

1. Virtual imaging simulation for hyper-hemi-microsphere


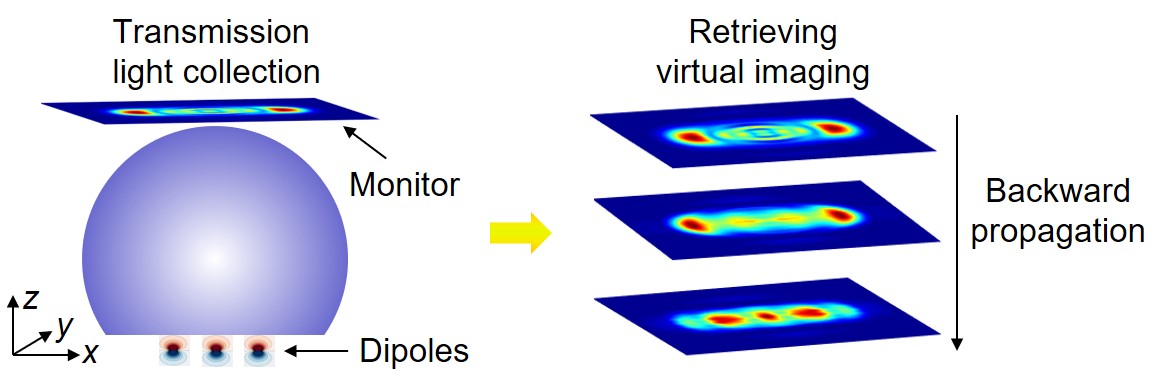


Fig. S1. Schematic of full-wave virtual imaging simulation for hyper-hemi-microsphere.

Full-wave virtual imaging simulations based on the principle of backward propagation are conducted to study the magnification of hyper-hemi-microsphere (HHMS) with different thicknesses as shown in Fig. S1. Limited by the memory of workstation, 2D full-wave virtual imaging simulations in the *xz* plane are conducted for HHMSs with different thicknesses. The virtual imaging simulations includes two parts. Firstly, the process of light from sample transmitting across the HHMS or microsphere boundaries is simulated with Lumerical FDTD software. The simulated transmission light field $\vec{E}_{t}(x)$ in a line 1 µm above the HHMS or microsphere is recorded for subsequent processing. The transmission light field $\vec{E}_{t}(x)$ is a vector, including two components $E_{\mathrm{tx}}$ and $E_{\mathrm{tz}}$. Secondly, the virtual image $I_{v}(x)$ is retrieved by the backward propagation of the recorded light field $\vec{E}_{t}(x)$. Based on the Fourier optics theory, if exp(-*iωt*) is taken as the form of the time dependence for the transmission light field, the virtual image $I_{v}(x)$ can be calculated by the following formulas in scalar field forms ^1-4^:

$E_{\text{tm}}\left(k_{\text{x}}\right)=\int_{-\infty}^{+\infty} E_{\text{tm}}\left(x\right)e^{\text{-}ik_{\text{x}}x}dx$, $k_{\text{x}}\leq\frac{2\pi}{\lambda}$, (S1)

$E_{\text{vm}}\left(k_{\text{x}}\right)=E_{\text{tm}}\left(k_{\text{x}}\right)e^{\text{-}i\Delta z\sqrt{k_{0}^{2}-k_{\text{x}}^{2}}}$, $k_{0}\text{=}\frac{2\pi}{\lambda}$ (S2)

$E_{\text{vm}}\left(x\right)= \frac{1}{2\pi}\int_{\text{-}k_{0}}^{k_{0}} E_{\text{vm}}\left(k_{\text{x}}\right)e^{ik_{\text{x}}x}dk_{\text{x}}$ (S3)

$I_{\text{v}}\left(x\right)=E_{\text{vx}}\left(x\right)E_{\text{vx}}\left(x\right)^{*}+E_{\text{vz}}\left(x\right)E_{\text{vz}}\left(x\right)^{*}$ (S4)

where $k_{x}$ and $\lambda$ are spatial frequency along the $x$ direction and wavelength, respectively. $E_{\mathrm{tm}}(k_{x})$ and $E_{\mathrm{vm}}(k_{x})$ are the spatial frequency spectra of the transmission light field in the collection and imaging planes, respectively. $\Delta z$ denotes the distance between the two planes. For virtual imaging, $\Delta z$ takes negative values to indicate the backward propagation. m represents different components of the light field vector and can indicate *x* and *z* directions in the 2D model. In calculations, the $E_{\mathrm{tx}}$ and $E_{\mathrm{tz}}$ component are substituted in equations S1-S3, respectively. The final light intensity $I_{v}(x)$ in virtual image is the sum of the calculation results from the two components.


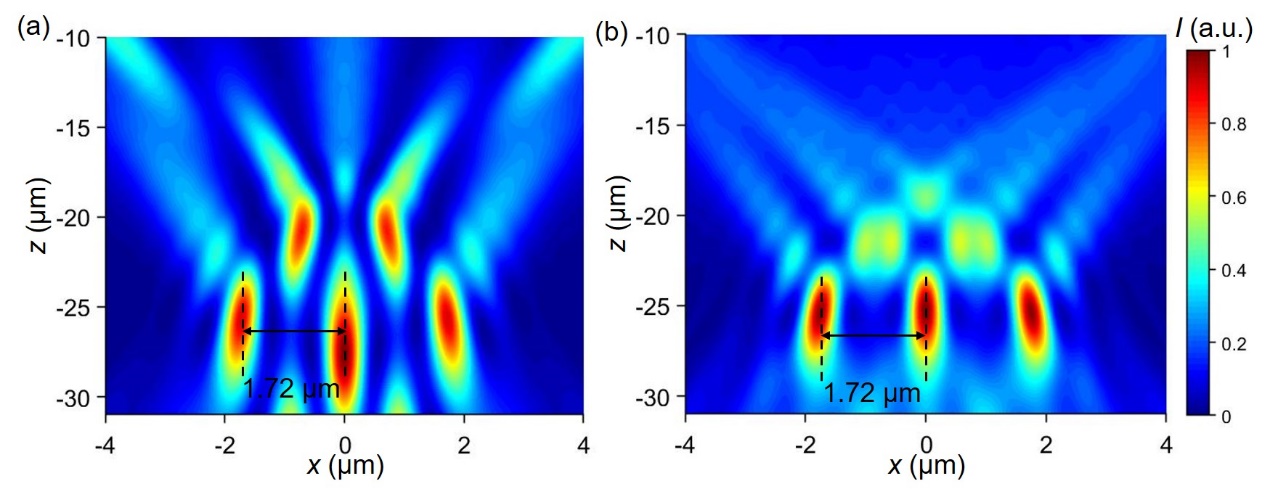


Fig. S2. 2D Full-wave virtual imaging simulation for three dipole sources. (a) simulating the virtual image of three coherent dipoles simultaneously and (b) simulating the virtual image of each dipole separately and then adding their virtual images together. *I*: normalized intensity.

The 2D full-wave virtual imaging simulations are conducted for HHMSs with different thicknesses. The same diameter of 24 μm is applied to all HHMSs (*n*=2.34). Imaging samples are three 300 nm-spaced electric dipoles oscillating along the *x* direction with respect to the coordinate system shown in Fig. S1. These dipoles are placed at *z*=0, which is the position of the flat plane of HHMSs. In the FDTD software, only coherent dipoles are available. Considering the possibility that coherence distorts the images, an alternative method to simulate the incoherent dipoles is calculating the virtual image $I_{v}(x)$ of each dipole separately and then adding the intensities $I_{v}(x)$ of the three images together to form an equivalent image of three incoherent dipoles. However, this alternative method is time consuming. By comparing imaging results acquired by this method and directly simulating three coherent dipoles, we find that the influence of coherence is limited for this simple case. Specifically, it does not affect our evaluation on the magnification of HHMSs, which is the main purpose to perform this simulation. As an example, the simulation of three 300 nm-spaced dipoles imaged by a HHMS (diameter 24 μm, height 17.2 μm and *n*=2.34) are conducted with the two methods. The imaging results are displayed in Fig. S2. The image is distorted a little due to the coherence of light when three dipoles are simulated simultaneously as shown in Fig. S2(a). However, the magnification evaluated from the center-to-center distance between the central and side dipoles are 5.73× for both methods. Therefore, it is acceptable that the coherent dipoles are used for imaging simulation to evaluate the magnification of HHMS with different thicknesses in this work.

The retrieved light field for HHMSs with different thicknesses *t*, i.e. 12 μm, 15 μm, 17 μm and 18.3 μm, is presented in Figs. S3(a)-(d). Imaging planes are indicated by white dashed lines. Magnification of the HHMSs increases with the thickness, for the four thicknesses it takes values of 2.4×, 3.3×, 5.3× and 10×, respectively.


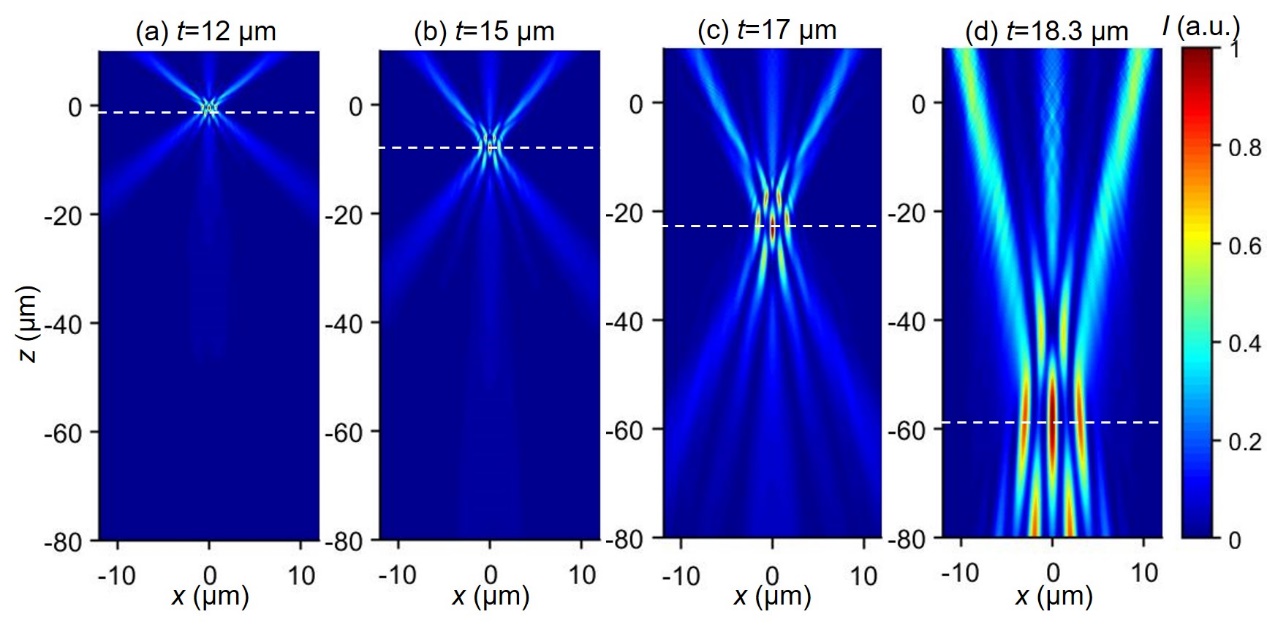


Fig. S3. Full-wave virtual imaging simulation for HHMSs with four thicknesses (a) $t=12 \mu m$, (b) $t=15 \mu m$, (c) $t=17 \mu m$ and (d) $t=18.3 \mu m$. *I*: normalized intensity.

2. Effective refractive index of microsphere


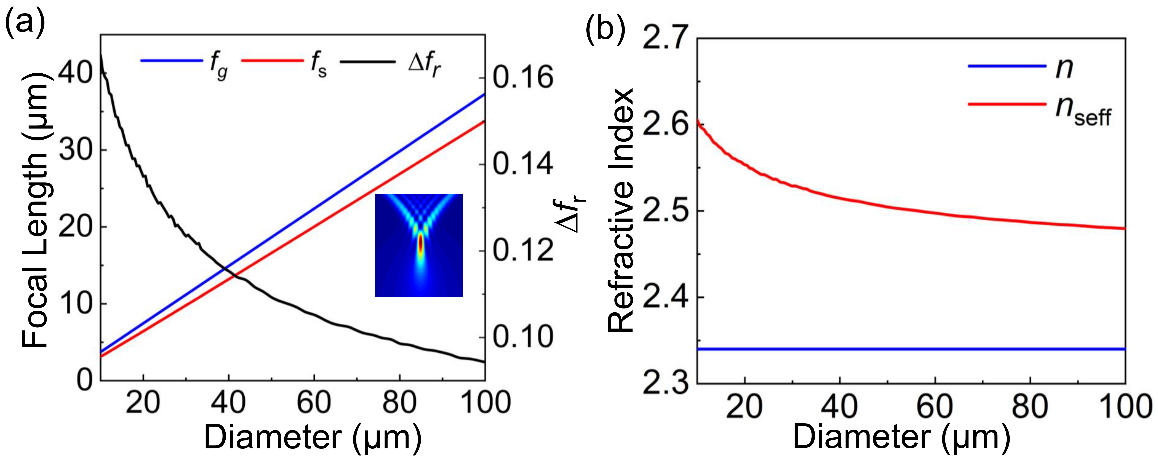


Fig. S4. (a) Focal length versus the diameter of BTG microsphere. Blue line is calculated with geometric optics formula. Red line is obtained from 2D FDTD simulation. The black plot indicates the relative ratio ${\Delta f}_{r}$ ${(\Delta f}_{r}=\left| f_{s}-f_{g} \right|/f_{s}$) of change in focal length between wave and geometric optics calculations. (b) Refractive index versus the diameter of BTG microspheres. Blue and red plots represent the nominal and effective refractive indices of BTG microspheres, respectively.

The focusing field of a microsphere with refractive index being larger than the critical refractive index is inside the microsphere. Simulations disclose that the focusing field of such microspheres are still photonic nanojets as displayed in the inset of Fig. S4(a). The simulation is conducted with the Lumerical FDTD software. Generally speaking, the point with the maximum intensity in the nanojet is utilized to estimate the focal length of a microsphere. The focal length measures the distance between the center of microsphere and the focal point. The simulated and calculated focal lengths $f_{s}$ and $f_{g}$ for barium titanate glass (BTG) microspheres (*n*=2.34) at 420 nm wavelength are presented in Fig. S4(a) with red and blue line plots, respectively. Since only the surface of the microsphere upon the focusing point contributes to the focusing field, the calculation of $f_{g}$ is based on a geometric optics formula for a single spherical surface as shown below (see Section 4.4 of ref. 5):

$f_{\text{g}}=\frac{n_{0}r}{n-n_{0}}$ (S5)

where $r$ is the radius, $n$ and $n_{0}$ the refractive indices of microsphere and environment, respectively. Similar to the situation of microsphere with refractive index being smaller than the critical refractive index, $f_{s}$ is smaller than $f_{g}$, which means a single micro-spherical surface still has stronger abilities to bend light beyond the predictions of geometric optics theory.

The previous research work of our group has reported using the effective refractive index of microsphere (calculated based on the focusing field outside microspheres) to modify the geometric optics theory for more accurate calculations ^6^. Here, for the case of microspheres with focusing field inside its body, a similar processing method can be adopted. Replacing $f_{g}$ with the simulated focal length $f_{s}$ in equation (S5), an equivalent refractive index value can be obtained. We call the equivalent refractive index value as “effective refractive index” and label it as $n_{\mathrm{seff}}$. The calculation formula for $n_{\mathrm{seff}}$ is presented in equation (3) in the main text. As an example, effective refractive indices of microspheres with *n*=2.34 are calculated and presented in Fig. S4(b) as the red curve. The effective refractive index $n_{\mathrm{seff}}$ increases as the diameter of the microsphere decreases, which is different from the nominal refractive index *n*. The $n_{\mathrm{seff}}$ has the same change trend as the relative ratio ${\Delta f}_{r}$ of change in focal lengths between wave and geometric optics calculations as shown in Fig. S4(a) the plotted black curve. The size effect on $n_{s\mathrm{eff}}$ and ${\Delta f}_{r}$ is reasonable since the wave optic effects become more prominent as the microsphere diameter is smaller and it can cause extraordinary bending of light. In the HHMS related optics design, the effective refractive index of microsphere associated with the modified theory presented in the main text provides more accurate predictions.

3. Numerical aperture of HHMS


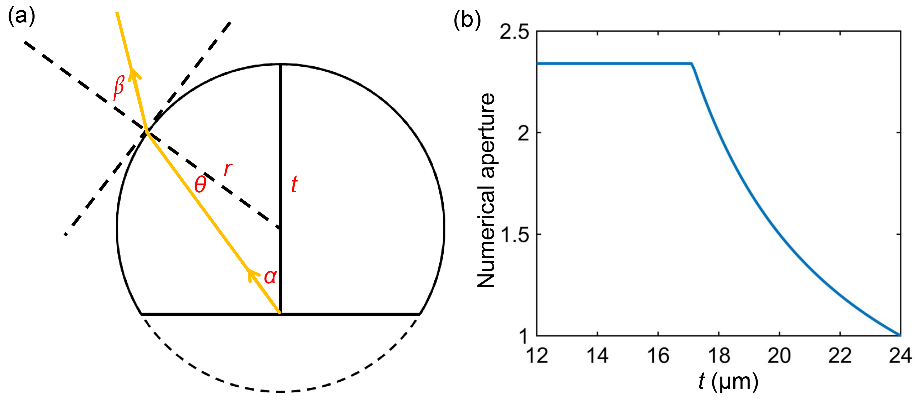


Fig. S5. (a) Schematic of light refraction in HHMS, (b) Numerical aperture of HHMS (*n*=2.34, *r*=12 μm) versus its thickness *t*.

The schematic of light refraction in HHMS is plotted in Fig. S5(a). If the thickness of HHMS increases, total internal reflection occurs for light with a large propagation angle $\alpha$, which limits the numerical aperture (NA) of HHMS. The critical thickness $t_{c}$ for total internal reflection of light emitting from the bottom surface of HHMS with 90° propagation angle can be calculated by:

$t_{\text{c}}=r+\frac{r}{n}$ (S6)

where *n* and *r* are the refractive index and radius of HHMS, respectively. When the thickness of HHMS is smaller than $t_{c}$, all light with propagation angles in range [-90° 90°] can exist the HHMS and thus the $\mathrm{NA}=n$. When the thickness of HHMS is larger than $t_{c}$, the propagation angle of light that can output from the HHMS should satisfy below requirement:

$\alpha\leq\arcsin\left[\frac{r}{n\left(t-r\right)}\right]$ (S7)

Therefore, the NA of HHMSs with thicknesses *t* can be calculated by:

$\text{NA=}\left\{\begin{matrix} n, \text{t}\leq t_{c} \\ \frac{r}{t-r}, \text{t > }t_{c} \end{matrix}\right.$ (S8)

The NA of HHMSs (*n*=2.34, *r*=12 μm) with different thicknesses are calculated with equation (S8) and displayed in Fig. S5(b). It can be found that when *t* > *t*_c_, NA and thickness are inversely proportional. Thus, we should balance the NA and magnification in the design of HHMS because the latter increases with the increase in thickness.

4. Comparison between HHMSs and liquid-immersed BTG microspheres


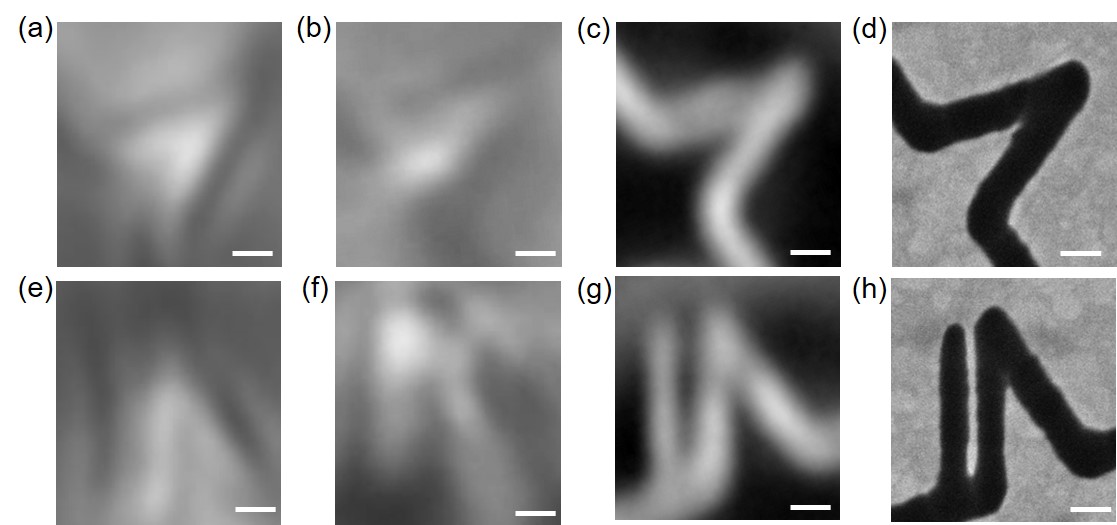


Fig. S6. Two angles of the star pattern imaged by (a) and (e) an 8 μm *n*~1.9 BTG microsphere immersed in *n*~1.34 oil, (b) and (f) an 8 μm *n*~2.2 BTG microsphere immersed in *n*~1.5 oil, (c) and (g) a BTG HHMS in air, and (d) and (h) SEM. Scale bars in (a)-(c) and (e)-(g) are 1 μm, and in (d) and (h) are 250 nm.

To further confirm the imaging performance of the 8 μm liquid immersed BTG microspheres, two other angles of the star pattern shown in Fig. 3(a) of the main text are also imaged by relocating the position of microspheres. The imaging results are presented in Fig. S6 and compared with images obtained by HHMS and SEM. Figs. S6(a) and (e) are acquired by the 8 μm *n*~1.9 BTG microsphere immersed in *n*~1.34 oil. Figs. S6(b) and (f) are obtained by 8 μm *n*~2.2 BTG microsphere immersed in *n*~1.5 oil. To facilitate the comparison, the virtual images of the two angles captured by HHMS are cropped from Fig. 3(f) in the main text and displayed in Figs. S6(c) and (g). Similarly, the SEM images of the two angles are cropped from Fig. 3(a) in the main text and displayed in Figs. S6(d) and (h). It is obvious that the image contrast of Figs. S6(c) and (g) is better than images captured by the two liquid immersed microspheres. In Figs. S6(e) and (f), the 54 nm gap between two lines is also discernible. However, the magnification on the gap is abnormal and the two images are distorted, which is consistent with the results shown in Fig. 3(d) and (e) in the main text. Fig. S6 further proves conclusions obtained from Fig. 3 in the main text, i.e. HHMS has higher imaging fidelity and contrast.

In addition, compared with liquid immersed microsphere, the magnification of HHMS can be easily customized by tailoring its thickness, which may also benefit the imaging resolution of HHMS assisted nanoscope. The full potential of imaging resolution of microsphere and HHMS can only be exhibited with sufficient magnification of the micro-optics. Even though the microsphere can provide sufficient resolution, if the magnification is low and the magnified feature size by the microsphere is still beyond the resolution limit of the subsequent objective lens, the high resolution of the microsphere is still not fully exploited to observe the tiny features. For example, if a feature with 50 nm size is imaged by a microsphere with 4× magnification, the magnified image of the feature is 200 nm, which is still hard to be resolved by a common 100× objective lens (NA~0.9) under white light illumination. High magnification of HHMS can be easily realized by increasing its thickness. In principle, the magnification of liquid-immersed microsphere can also be enlarged by selecting proper immersion medium to increase relative refractive indices of microsphere and immersion medium according to the formula below ^7^:

$M\left(n'\text{, }r\text{, }g\right)=\frac{n'}{2\left(n'-1\right)\left(\frac{g}{r}+1\right)-n'}$ (S9)

where *r* is the radius of the microsphere, $n^{'}$ the relative refractive index between the microsphere and object space, and *g* the gap. If the BTG microspheres (*n*=2.34) touch the sample and are immersed in a commonly available liquid, such as water (*n*=1.34) or ethanol (*n*=1.37), the magnification $\left| M \right|$ is 6.9× and 5.9×, respectively. If 10× magnification is required for the BTG microsphere (*n*=2.34) working in contact mode, the refractive index of immersion medium should be 1.287, which is hard to get such a low refractive index liquid. To develop a special immersion liquid for the high refractive index BTG microsphere may also be complex and expensive. Besides, the numerical aperture and magnification of liquid-immersed BTG microspheres should be balanced by selecting proper liquid medium since the change of liquid medium’s refractive index has contradicting effects on the two factors. Therefore, there are limitations on increasing the magnification of liquid-immersed microsphere by only changing the immersion medium.

To summarize, compared with 4-10 μm liquid-immersed BTG microspheres, HHMS shows several advantages, including high imaging fidelity, high imaging contrast, big field-of-view and easily customized magnification. In terms of the high cost on the fabrication of HHMS, it may be solved by adopting other fabrication methods, such as high temperature molding.

5. Comparisons among different methods to increase magnification

Except for the HHMS, there are other reported methods that can increase the magnification, such as using microspheres compound lens or sphere with refractive index near the critical value ^6,8,9^. The microspheres compound lens can increase magnification whereas it cannot promote the resolution beyond the bottom microsphere. The main advantage of the microspheres compound lens is that it allows the use of low magnification objective lens for outstanding optical nano-imaging at low cost. The overall resolution of the microsphere compound lens is still restricted by the resolution and magnification of the bottom microsphere. In brief, on the basis that the bottom microsphere has a high resolution, it should also have sufficient magnification to make the magnified feature size of sample within the resolution limit of the upper microsphere in the compound lens. Otherwise, the full imaging resolution potential of the bottom microsphere cannot be completely exploited, and the imaging system resolution is still restricted. In comparison, the large NA of the high refractive index HHMS promises the lens with a high resolution. The high magnification of HHMS can help the lens to exhibit its full potential of imaging resolution. Therefore, although both the compound lens and the HHMS possess high magnification, the effect of increased magnification on imaging resolution is different.

Recently, two works reported that 50× magnification realized by ball lenses ^8,9^. They present impressive experimental results that using ball lens with refractive index near the critical value to achieve ultra-high magnification and the diffraction-limited resolution by only coupled with a smartphone. This simple, low-cost but competent imaging technique promises great application potentials. Comparing this method with the HHMS, one advantage of the HHMS is that its magnification can be easily tuned by tailoring its thickness as shown in Fig. 1(d) and Fig. 4 in the main text. Although the highest magnification of HHMS presented in the manuscript is 10×, there is no obvious difficulty to achieve 50× magnification or larger. In this method, the high magnification attributes to the use of critical refractive index, which is determined by the material of the lens and its surrounding environment. To our knowledge, it is not easy to change the refractive index of material. So, once the material of lens is determined, its magnification in a certain immersion medium is also fixed and cannot be tuned at will. The other advantage of HHMS is that the HHMS made of high refractive index material can achieve a large magnification and high numerical aperture simultaneously. Restricted by the critical refractive index requirement for high magnification, the environment refractive index should be around half of the ball lens refractive index. The ball lens’ numerical aperture cannot be close to its own high refractive index value, thus the imaging resolution is limited. Certainly, the proposed ball lens in these papers possess advantages in other aspects, such as low-cost and large field-of-view. Both the HHMS and ball lens can leverage their strengths in different application scenarios.

6. Design of HHMS composed compound lens


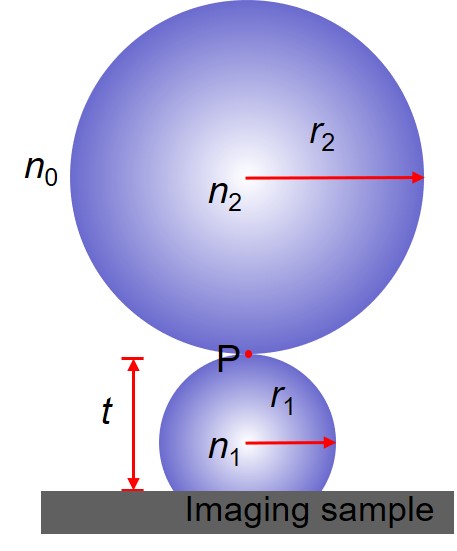


Fig. S7. Schematic of hyper-hemi-microsphere composed compound lens.

The schematic of hyper-hemi-microsphere composed compound lens (HHMS-CL) for nano-imaging is presented in Fig. S7. According to the geometric optics theory (see Section 4.4 of ref.5), the imaging distance (position) and magnification of a single HHMS can be calculated by equations (S10) and (S11), respectively.

$l_{1}^{'}=\frac{n_{0}ar_{1}}{\left(n_{1}-n_{0}\right)a-n_{1}}$ (S10)

$\beta_{1}=\frac{n_{1}}{\left(n_{0}-n_{1}\right)a\text{+}n_{1}}$ (S11)

The derivation of equations (S10) and (S11) are based on the relationship between object and image distances for a single spherical surface. Equations (S10) and (S11) describe the case when the object touches the HHMS. Where $l_{1}^{'}$ is the image distance. The origin point used for measuring the object distance and image distance is the pole P of the spherical surface of the HHMS. If the image locates at the bottom side of the origin, $l_{1}^{'}$ takes a negative value. Otherwise, it takes a positive value. $\beta_{1}$ is the magnification factor. $r_{1}$ and *t* are the radius and thickness of the HHMS, respectively. $a=t/r_{1}$. *n*_0_ and *n*_1_ are the refractive indices of environment and HHMS, respectively.

In the HHMS-CL, the upper microsphere takes the image produced by the bottom HHMS as the sample and image it again. The origin point used for measuring the object distance and image distance of the upper microsphere is the same as that of the HHMS, i.e. the point P in Fig. S7. As such, the image distance $l_{2}^{'}$ and magnification 𝛽_2_ of the upper microsphere can be calculated by equations (S12) and (S13) as following:

$l_{2}^{'}=\frac{\left(2n_{0}\text{-}n_{2}\right)r_{2}l_{1}^{'}-2n_{0}r_{2}^{2}}{2\left(n_{2}\text{-}n_{0}\right)l_{1}^{'}+2n_{0}r_{2}-n_{2}r_{2}}\text{+}2r_{2}$ (S12)

$\beta_{2}=\frac{n_{2}r_{2}\left[\left(n_{1}-n_{0}\right)a-n_{1}\right]}{2\left(n_{2}\text{-}n_{0}\right)n_{0}ar_{1}+r_{2}\left(2n_{0}-n_{2}\right)\left[\left(n_{1}-n_{0}\right)a-n_{1}\right]}$ (S13)

where *n*_2_ and *r*_2_ are the refractive index and radius of the upper microsphere, respectively. The total magnification 𝛽_HHMCL_ of the HHMS-CL can be calculated by equation (S14) as following:

$\beta_{\text{HHMCL}}=\beta_{1}\times\beta_{2}=\frac{n_{1}n_{2}r_{2}}{2\left(n_{0}\text{-}n_{2}\right)n_{0}ar_{1}\text{+}\left(2n_{0}-n_{2}\right)\left[\left(n_{0}-n_{1}\right)a\text{+}n_{1}\right]r_{2}}$ (S14)

When the upper microsphere works near the boundary between real and virtual imaging modes, $\beta_{\mathrm{HHMCL}}\to\infty$. The radius ratio of the microsphere and HHMS should satisfy:

$\frac{r_{2}}{r_{1}}=\frac{2n_{0}a\left(n_{2}-n_{0}\right)}{\left[a\left(n_{1}-n_{0}\right)-n_{1}\right]\left(n_{2}-2n_{0}\right)}\text{=}k_{1}$ (S15)

When the magnification 𝛽_HHMCL_ of the HHMS-CL is -1, the radius ratio of the microsphere and HHMS should satisfy:

$\frac{r_{2}}{r_{1}}=\frac{2an_{0}\left(n_{2}-n_{0}\right)}{2n_{0}n_{1}+a\left(n_{0}-n_{1}\right)\left(2n_{0}-n_{2}\right)}\text{=}k_{2}$ (S16)

Hence, the condition for the HHMS-CL working in virtual-real imaging mode (i.e. the bottom HHMS works in virtual imaging mode and the upper microsphere works in real imaging mode) and remaining magnifying function is:

$k_{1}>r_{2}/r_{1}>k_{2}$ (S17)

where *k*_1_ and *k*_2_ are the two boundaries. Specifically, if the upper microsphere is designed to enlarge the field-of-view in real imaging and does not change the magnification of the whole system, the magnification 𝛽_2_ is -1. The radius ratio of the microsphere and HHMS should satisfy:

$\frac{r_{2}}{r_{1}}=\frac{a\left(n_{2}-n_{0}\right)}{n_{1}-a\left(n_{1}-n_{0}\right)}$ (S18)

Significantly, if we substitute the refractive indices of material with the effective refractive indices of microsphere and HHMS in the above equations, we can calculate the related parameters with higher precisions.

References

1 Duan, Y. B., Barbastathis, G. and Zhang, B. L. Classical imaging theory of a microlens with super-resolution. *Optics* *Letters* **38**, 2988-2990 (2013).

2 Sundaram, V. M. & Wen, S. B. Analysis of deep sub-micron resolution in microsphere based imaging. *Applied Physics Letters* **105**, 204102 (2014).

3 Maslov, A. V. & Astratov, V. N. Imaging of sub-wavelength structures radiating coherently near microspheres. *Applied Physics Letters* **108**, 051104 (2016).

4 Maslov, A. V. & Astratov, V. N. Resolution and reciprocity in microspherical nanoscopy: point-spread function versus photonic nanojets. *Physical Review Applied* **11**, 064004 (2019).

5 Born, M. & Wolf, E. Principles of optics: electromagnetic theory of propagation, interference and diffraction of light. (Elsevier, 2013).

6 Wu, G. X. & Hong, M. H. Optical nano-imaging via microsphere compound lenses working in non-contact mode. *Optics Express* **29**, 23073-23082 (2021).

7 Allen, K. W. *et al.* Super-resolution microscopy by movable thin-films with embedded microspheres: resolution analysis. *Annalen der Physik* **527**, 513-522 (2015).

8 Astratov, V. N. *et* *al*. Ball lens-assisted smartphone microscopy with diffraction-limited resolution. Proceedings of SPIE 12152, Mesophotonics: Physics and Systems at Mesoscale. Strasbourg, France: SPIE, 2022, 31-36.

9 Jin, B. Y. *et* *al*. Label-free cellphone microscopy with submicron resolution through high-index contact ball lens for *in* *vivo* melanoma diagnostics and other applications. Proceedings of SPIE 11972, Label-free Biomedical Imaging and Sensing. San Francisco, California, United States: SPIE, 2022, 107-111.
